# Study on the Effect of Substitutional Doping of Ce Atomic on the Damage Properties of Fused Silica

**DOI:** 10.3390/ma19061225

**Published:** 2026-03-20

**Authors:** Jiaxing Chen, Kaizao Ni, Ruijin Hong, Lingqiao Li, Zhan Sui

**Affiliations:** 1 School of Optical-Electrical and Computer Engineering, University of Shanghai for Science and Technology, No. 516, Jungong Road, Shanghai 200093, China; chenjiaxing@siom.ac.cn; 2 Shanghai Institute of Optics and Fine Mechanics, Chinese Academy of Sciences, No. 899, Huiwang East Road, Jiading District, Shanghai 201815, Chinallq@siom.ac.cn (L.L.); 3 Shanghai Institute of Laser Plasma, China Academy of Engineering Physics, 197 Chengzhong Road, Jiading District, Shanghai 201800, China; lqling@vip.163.com

**Keywords:** fused silica, cerium doping, energy band structure, impurity defects

## Abstract

In high-power laser systems, extrinsic impurities—particularly Ce introduced during conventional ring polishing—have been identified as critical contributors to the degradation of laser-induced damage resistance in fused silica optical components. This study systematically investigates the effects of cerium substitutional doping on the electronic structure and optical properties of fused silica, integrating first-principles density functional theory calculations with experimental characterizations. The results demonstrate that substitutional incorporation of cerium atoms into the fused silica framework introduces deep-level defect states within the band gap, resulting in band gap narrowing and absorption edge redshift of the material. The energy position of the defect states depends on the Ce doping configuration. Among them, the Ce-4f orbital constitutes the dominant component of the defect state’s electronic structure, while the neighboring atomic orbitals such as O-2p and Si-3s/3p participate in bonding through hybridization, thereby determining the depth and distribution characteristics of the defect levels. The optical absorption edge of cerium-doped fused silica undergoes a significant redshift from the intrinsic value of 222 nm to 468 nm in the dual-Ce adjacent-site doping configuration, thereby endowing the material with substantial optical absorption capability at a wavelength of 355 nm. μ-UVPL spectroscopy combined with μ-XRD and other characterization analyses confirmed that the characteristic emission peak at 450 nm on the surface region of fused silica originated from Ce-related defect centers; this spectral feature was consistent with the defect state electronic structure predicted by the diatomic nearest-neighbor doping model. LIDT tests further indicated that the Ce-contaminated area significantly weakened the material’s laser damage resistance under 355 nm laser irradiation. This study further explained the mechanism by which traditional polishing-induced Ce element doping causes the low laser damage threshold of fused silica optical components, providing a theoretical basis for improving their performance.

## 1. Introduction

Fused silica glass is widely employed in high-power laser optical systems owing to its exceptional optical performance, low thermal expansion coefficient, and excellent stability under ultraviolet (UV) irradiation. However, during precision machining processes, the surface and subsurface layers of fused silica components are prone to introducing structural and chemical defects such as microcracks, residual stresses, amorphized regions, and organic/inorganic impurities. Under intense UV laser irradiation, these defects can act as optical absorption centers or damage initiation sites, significantly reducing the components’ practical laser-induced damage threshold (LIDT). This leads to irreversible damage at fluences far below the theoretical intrinsic damage threshold, which not only restricts the long-term operational reliability of optical systems but also substantially drives up the costs of system maintenance and component replacement.

Numerous researchers have conducted extensive investigations into the laser-induced damage performance of fused silica. Weiyuan Luo et al. [1] demonstrated that oxygen ion implantation, which generates an oxygen-rich environment, effectively mitigates oxygen-deficient centers (ODCs) and E’ center defects in fused silica, enhances surface compactness, and thereby improves the material’s resistance to laser-induced damage. Zhichao Liu et al. [2] employed the artificial indentation method to examine cracks and brittle fractures in fused silica, revealing that such structural features contain a variety of point defects responsible for increased photoluminescence and photothermal absorption. Their study established a correlation between the intrinsic characteristics of indentations and the laser damage threshold, indicating that the high concentration of point defects within brittle cracks and fracture surfaces serves as a primary initiation site for laser damage. Shen Xiong et al. [3] investigated the damage behavior of optical glass components under laser plasma exposure, providing further insight into the underlying damage mechanisms. For such components, laser-induced ionization breakdown constitutes the dominant failure mechanism; in particular, shock waves generated by laser plasma can induce large-scale fracture damage in fused silica. Research on this fracture process has shown that the plasma-induced shock wave exerts compressive stress, resulting in layered fracture morphology at damage sites. Furthermore, the interference between reflected and incident waves produces tensile stress, promoting radial crack propagation in the glass matrix.

The aforementioned studies have primarily focused on the intrinsic defects of fused silica and processing-induced structural damage, providing detailed insights into the mechanisms by which these native imperfections initiate and propagate laser-induced damage in fused silica glass. However, in recent years, extrinsic impurities introduced during manufacturing—recognized as a significant contributor to laser damage—have increasingly drawn attention within the scientific community. In 2012, Libin Wang et al. [4] reported that under triple-frequency (355 nm) laser irradiation, metallic contaminants such as Al, Fe, and Cu induce damage in fused silica through extreme thermal and mechanical stresses resulting from rapid vaporization or micro-explosion. In 2017, Hongxiang Wang et al. [5] demonstrated that surface-bound impurities significantly reduce the laser damage threshold, while subsurface defects act as scattering centers that modulate the incident optical field. This modulation leads to localized constructive interference, thereby enhancing the electric field intensity, promoting multiphoton ionization, and initiating high-energy plasma formation, ultimately culminating in material ablation. Jian Cheng et al. [6] applied finite element analysis to model heat conduction and thermoelastic responses during laser energy deposition, identifying CeO_2_ nanoparticles as the most detrimental impurity species degrading the laser damage resistance of fused silica. Experimental results showed that HF acid etching, used to remove the redeposited layer formed during polishing, reduced CeO_2_ defect density by approximately 50%, leading to an improved damage threshold. Nevertheless, residual Ce-containing species were still detected after treatment, indicating incomplete removal of deeply embedded impurities. Although the ammonium bifluoride-based aqueous etching process (AMP), developed by Tayyab I. Suratwala et al. [7], has been proven effective in increasing the surface laser damage threshold and is currently implemented at the U.S. National Ignition Facility, its limited penetration depth restricts efficacy in removing absorption-inducing defects located within narrow lateral cracks. Xiang Gao et al. [8] developed a probabilistic model for surface damage initiation in fused silica optical components based on Mie scattering theory and transient heat conduction equations. Their simulations indicated that under 355 nm laser irradiation, CeO_2_ particles contribute less to damage probability compared to Al and Cu inclusions, primarily due to differences in optical absorption cross-section and thermal response. Li et al. [9,10] employed argon ion beam sputtering coupled with depth-resolved profiling to perform controlled layer-by-layer removal of the fused silica surface, enabling precise characterization of the spatial distribution of impurity elements, subsurface defects, and their corresponding laser-induced damage thresholds (LIDTs). The study revealed that metallic impurities exhibit pronounced broadband optical absorption, which triggers intense localized temperature elevation and accumulation of thermomechanical stress. Furthermore, polishing residues were confirmed to induce the formation of point defects—including oxygen-deficient centers (ODCs) and non-bridging oxygen hole centers (NBOHCs)—accompanied by the incorporation of metallic impurities such as Ce, Fe, and Al. These defect-impurity composite structures are widely recognized as core precursors to laser-induced damage, with their spatial distribution extending to depths greater than 2 μm below the surface. In summary, processing-induced impurities retained within the subsurface region of fused silica—particularly cerium-containing species—have been identified as critical contributors to the significant reduction in its LIDT across the UV and deep UV spectral regimes. While existing studies have preliminarily established correlations between the spatial distribution of impurities and macroscopic laser damage responses, the atomic-scale interaction mechanisms between these impurities and high-energy photons remain poorly understood. Systematic investigations into the underlying microscopic mechanisms are therefore urgently warranted.

In this paper, the potential mechanism underlying the degradation of laser damage resistance in fused silica induced by residual Ce impurities following chemical mechanical polishing (CMP) is elucidated via a synergistic approach combining molecular dynamics (MD) simulations and first-principles calculations. By constructing fused silica models doped with single and dual Ce atoms, computational analyses of band structures, electronic density of states (DOS), and absorption spectra reveal that Ce impurities introduce localized defect states within the fused silica, resulting in bandgap narrowing and absorption edge redshift. This effect enhances the absorption of 355 nm laser radiation, which constitutes a key driver of laser-induced damage. Experimental characterizations, including photothermal weak absorption (PTWA), micro-ultraviolet photoluminescence (μ-UVPL), micro-X-ray diffraction (μ-XRD), and micro-X-ray fluorescence (μ-XRF), demonstrate that the ~450 nm fluorescence is associated with amorphous Ce-related defects, consistent with the luminescence peak of Ce-doped glass. LIDT measurements further confirm that regions containing Ce impurities exhibit a significantly reduced damage threshold, thus identifying them as primary damage initiation sites. This study bridges atomic-scale electronic structures, micrometer-scale defects, and macroscopic damage performance, and clarifies the relationship between residual Ce from CMP and the degradation of laser damage characteristics in ultraviolet high-power laser optical components.

## 2. Materials and Methods

### 2.1. Model Construction

This study investigates Ce atom doping in fused silica, which occurs during the ring polishing process. CeO_2_, commonly used as an abrasive, undergoes interfacial chemical reactions with the fused silica surface, promoting the incorporation of Ce atoms into the surface and subsurface regions. The underlying reaction mechanism is described as follows:

(1) $$\equiv Ce\mp H_{2}O↔\equiv Ce–OH+H^{+}$$

(2) $$\equiv Si–O+H_{2}O↔\equiv Si–OH+OH^{-}$$

(3) $$\equiv Si–OH+HO–Ce\equiv ↔\equiv Si–O–Ce\equiv +H_{2}O$$

As reported by Fukun Li et al. [11], in an aqueous polishing environment, the surfaces of CeO_2_ abrasive particles and fused silica glass undergo hydroxylation to form Ce–OH and Si–OH functional groups, respectively. During mechanical polishing, these hydroxylated species migrate into the near-surface region of fused silica. Under applied abrasive pressure or interfacial shear stress, they react through a condensation process to form stable Ce–O–Si bridging bonds, effectively anchoring Ce atoms within the silica network. This bonding process arises from the coordination of lone-pair electrons from deprotonated ≡Si–O^−^ sites into the empty 5d orbitals of Ce^3+^, resulting in Ce–O–Si bonds with both coordination and partial covalent character. Despite local electron redistribution, the system maintains overall charge conservation and local electrical neutrality.

Notably, fused silica optical components for high-power laser applications undergo multiple post-treatment steps, including CMP and acid washing. During these processes, free Ce, Ce oxide clusters, and large Ce-containing impurities are preferentially removed due to their low chemical stability or weak physical adhesion. In contrast, Ce^3+^ embedded in the SiO_2_ network via Ce–O–Si covalent bonds exhibits higher thermodynamic and chemical stability, making it more resistant to removal by subsequent processes. Therefore, this study focuses on such structurally stable substitutional Ce defects, constructing atomic-scale models to systematically investigate the effects of residual Ce impurities on the intrinsic electronic structure and optical properties of fused silica. The specific modeling strategy involves substituting tetrahedrally coordinated Si^4+^ sites with Ce^3+^ in a disordered fused silica structure, followed by structural relaxation and energy minimization to obtain thermodynamically stable Ce–O–Si bonding configurations.

### 2.2. Construction of the Pristine Fused Silica Model

**Figure 1:**
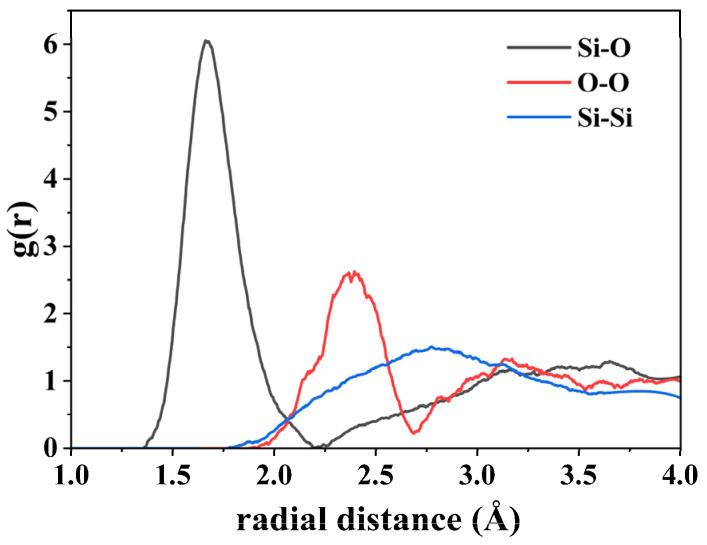
RDF of the Amorphous Fused Silica Structure Constructed via MD Simulation.

**Figure 2:**
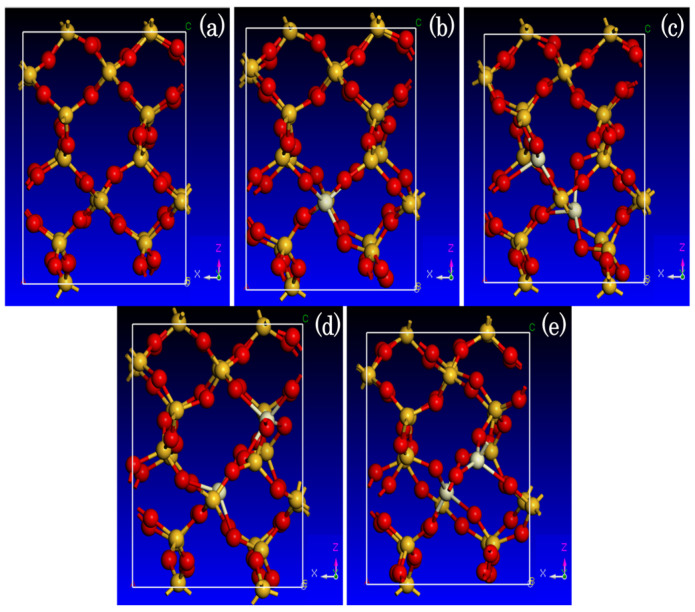
Atomic models of intrinsic fused silica and Ce-substitutional doping configurations. Si atoms: yellow; O atoms: red; Ce atoms: white. (**a**) Intrinsic fused silica model; (**b**) Vertex-site substitutional doping model; (**c**) Adjacent-site substitutional doping model; (**d**) Shoulder-site substitutional doping model; (**e**) Opposite-site substitutional doping model.

The amorphization of crystalline quartz into fused silica was achieved through MD simulations using the LAMMPS (stable_23Jun2022) software package and the classical Tersoff potential. A three-step thermal annealing protocol was implemented to simulate the melting-quenching process, resulting in a fully amorphous structure, as illustrated in Figure 2a. The initial configuration consisted of a β-quartz unit cell containing 75 atoms (25 Si and 50 O), with lattice constants (a = 8.677 Å, b = 5.010 Å, and c = 5.470 Å). Periodic boundary conditions were applied in all spatial directions to represent bulk behavior. Simulations were conducted under the NVT ensemble using the Nosé–Hoover thermostat. The protocol included the following stages: The system was heated from 300 K to 6000 K at a rate of 0.6 K/ps over 10 ps to ensure complete disruption of the crystalline lattice; The system was held at 6000 K for 50 ps to eliminate residual structural order; The molten sample was cooled to 300 K at a rapid rate of 570 K/ps over 10 ps to form an amorphous network, followed by a 50 ps equilibration at 300 K to achieve structural stability. All simulations employed a time step of 1 fs. The Tersoff potential parameters were taken from ref. [12], with a cutoff distance of 2.0 Å for Si–O interactions to accurately capture short-range bonding characteristics. This potential has been extensively validated in previous MD studies of fused silica and is well established for modeling its structural and thermodynamic properties, thereby ensuring the robustness of the generated amorphous model. The intrinsic amorphous fused silica model constructed in this study was validated. The density of the simulated amorphous structure was determined to be 2.23 g/cm^3^, which aligns well with the experimentally reported bulk density of fused silica [13]. Figure 1 presents the radial distribution function (RDF) of the model, where the first Si–O coordination peak is centered at approximately 1.67 Å, corresponding to the characteristic covalent Si–O bond length. The O–O correlation peak is observed at around 2.30 Å, while the Si–Si correlation peak appears at approximately 2.75 Å—these characteristic peak positions are consistent with the experimental data from previously published MD models of fused silica [14]. Integrating the density matching and RDF structural features, the results demonstrate that the amorphous model established in this work exhibits excellent physical rationality and structural fidelity at the atomic scale, making it a reliable initial configuration for subsequent first-principles investigations into electronic structures and optical properties.

### 2.3. Construction of Ce-Substitutional Doping Models with Distinct Configurations

To investigate the influence of Ce atom positioning on the electronic structure, substitutional doping was implemented within the Si–O six-membered ring of fused silica by replacing one or two Si atoms with Ce atoms, while preserving the total number of atoms. Four distinct doping configurations were systematically examined based on the relative positions of the dopants:

(1) Vertex-site substitutional doping (Figure 2b): A single Ce atom substitutes a Si atom at a vertex site of the six-membered ring;
(2) Adjacent-site substitutional doping (Figure 2c): Two Ce atoms replace adjacent Si atoms in the ring;
(3) Shoulder-site substitutional doping (Figure 2d): Two Ce atoms occupy sites separated by one intervening Si atom;
(4) Opposite-site substitutional doping (Figure 2e): Two Ce atoms substitute Si atoms located at opposite (diagonal) positions within the ring.

All doped structures underwent full structural relaxation until the maximum residual atomic force fell below 0.05 eV/Å, ensuring energetic convergence. In the computational model of this study, Ce atoms form Ce–O–Si bridge bonds with adjacent oxygen atoms, which exhibit both coordinative and covalent character. Despite the limited system size, spin polarization effects were incorporated. Given the strong correlation of Ce-4f electrons, to enhance the accuracy of electronic structure calculations, the HSE06 hybrid functional with spin–orbit coupling (SOC) was employed to more precisely describe electron localization. Furthermore, prior studies have demonstrated that the HSE06 hybrid functional yields predictions of lanthanide f-electron properties comparable to those from GW0 and LDA + U calculations [15,16]. Plane-wave ultrasoft pseudopotentials were utilized, with the plane-wave kinetic energy cutoff set to 700 eV, and Brillouin zone integration was performed using a Γ-centered 1 × 1 × 1 k-point grid. Self-consistent field (SCF) iterations were converged to a tolerance of 1 × 10^−6^ eV per atom. The band gap of the pristine fused silica model, constructed from the LAMMPS-generated structure (Section 2.2), was calculated to be 7.532 eV—slightly lower than the experimental value. This underestimation is consistent with known limitations of the HSE06 functional in accurately predicting absolute band gaps for wide-bandgap insulators. Nevertheless, as this study emphasizes the relative variation in band gap upon Ce doping rather than absolute band gap values, the qualitative trends and comparative analysis remain valid and unaffected.

### 2.4. Experimental Methods

To validate the influence of defect states—predicted by the fused silica impurity model—on the optical and structural properties of fused silica glass, a series of experimental characterizations were conducted following sample preparation. The procedures are summarized as follows. All fused silica samples employed in this study were supplied by Shanghai Hengyi Optical Precision Machinery Co., Ltd. (Shanghai, China), and subjected to multiple cycles of synergistic annular polishing and acid cleaning processes. Their nominal dimensions are 20 mm × 20 mm × 5 mm. Prior to measurement, the samples were subjected to ultrasonic cleaning in deionized water for 15 min.

The PTWA values and positional coordinates of the defective regions were acquired using a small-aperture photothermal weak absorption tester. A 355 nm wavelength laser served as the pump beam, while a 632 nm wavelength laser was employed as the probe beam. With a single-point aperture of 20 μm × 20 μm, the fused silica glass sample was subjected to point-by-point scanning. UV fluorescence spectra of defects in fused silica glass samples were characterized using a micro-UV fluorescence spectrometer, with an excitation wavelength set at 355 nm. Distinct fluorescence phenomena and corresponding spectral features were observed. Subsequently, the elemental composition of surface fluorescent defects was analyzed via micro-XRF (Horiba XGT-9000 X, Kyoto, Japan, The instrument was calibrated against the manganese Kα characteristic X-ray line (Mn Kα = 5.899 keV). The accelerating voltage of the X-ray tube was set to 50 kV, and the tube current was 550 μA) and micro-XRD (Bruker D8 Discover, Billerica, MA, USA, The measurement was performed under the following conditions: Cu Kα radiation, 50 kV accelerating voltage, and 1000 μA tube current), where the diameters of the detection areas were 400 μm and 200 μm, respectively. Finally, the laser damage threshold (LDT) of the fluorescent defect regions in the samples was determined.

## 3. Results and Discussion

### 3.1. Effects of Ce Doping on the Electronic Structure of the Intrinsic Fused Silica Model

Following substitutional doping of the pristine fused silica model with one or two Ce atoms at Si sites within the Si–O six-membered ring, the bond lengths and bond angles between Ce atoms and their adjacent oxygen atoms are summarized in Table A1.

**Figure 3:**
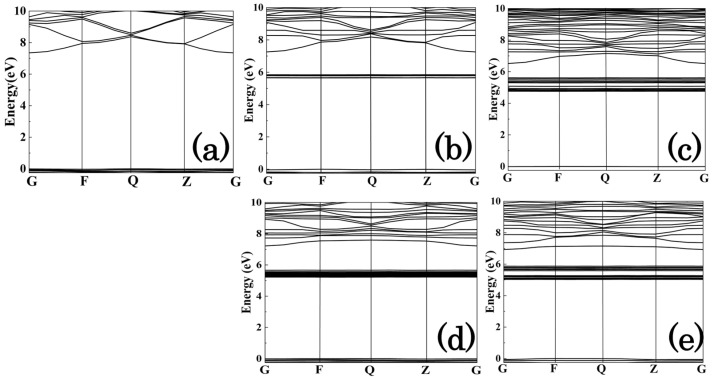
Band Structures of Intrinsic Fused Silica and Ce-Substitutional Doping Models: (**a**) intrinsic fused silica; (**b**) vertex substitutional doping; (**c**) adjacent-site substitutional configuration; (**d**) shoulder substitutional configuration; (**e**) opposite substitutional configuration.

Using the optimized geometric structures, the electronic band structures of the intrinsic fused silica model and Ce substitution doping model—with one or two Ce atoms replacing Si atoms in the Si–O six-membered ring—were calculated within the framework of DFT using the HSE06 hybrid functional. The results are presented in Figure 3. As shown in the figure, the band gaps of all cerium-doped models are narrower than that of the intrinsic fused silica model, and defect bands consisting of multiple discrete defect states emerge within the band gap region of the intrinsic fused silica.

**Figure 4:**
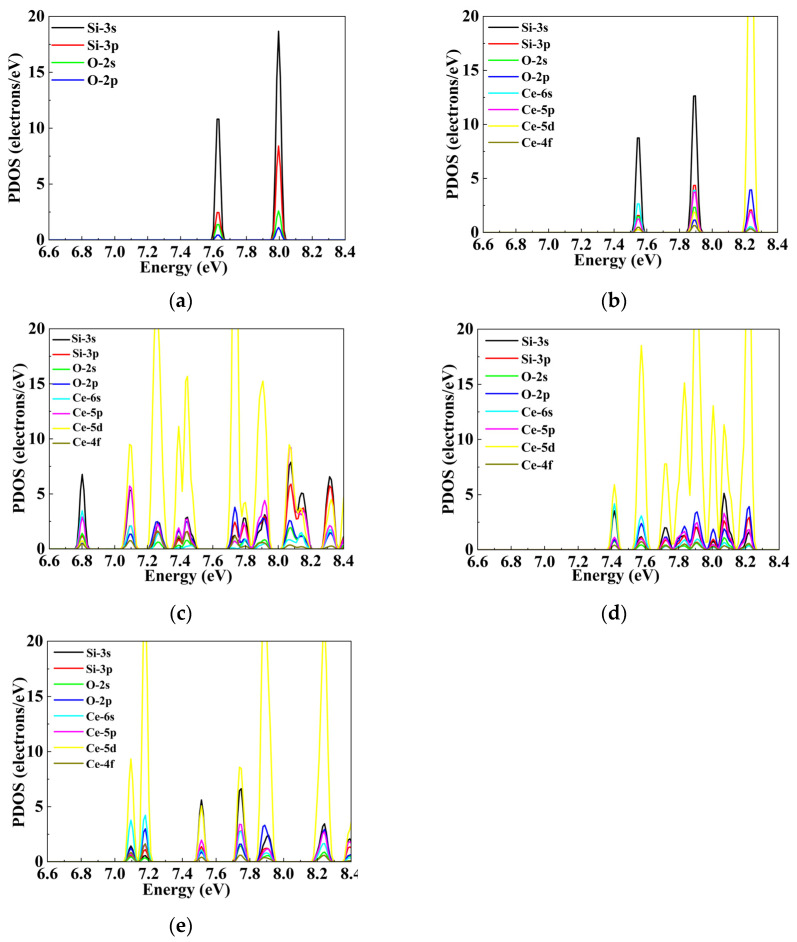
Comparison of PDOS at the CBM Between Intrinsic Fused Silica Model and Ce-Substitutional Doped Fused Silica Model: (**a**) intrinsic fused silica; (**b**) Ce vertex substitutional doping; (**c**) Adjacent-site substitutional configuration; (**d**) shoulder substitutional configuration; (**e**) opposite substitutional configuration.

**Figure A1:**
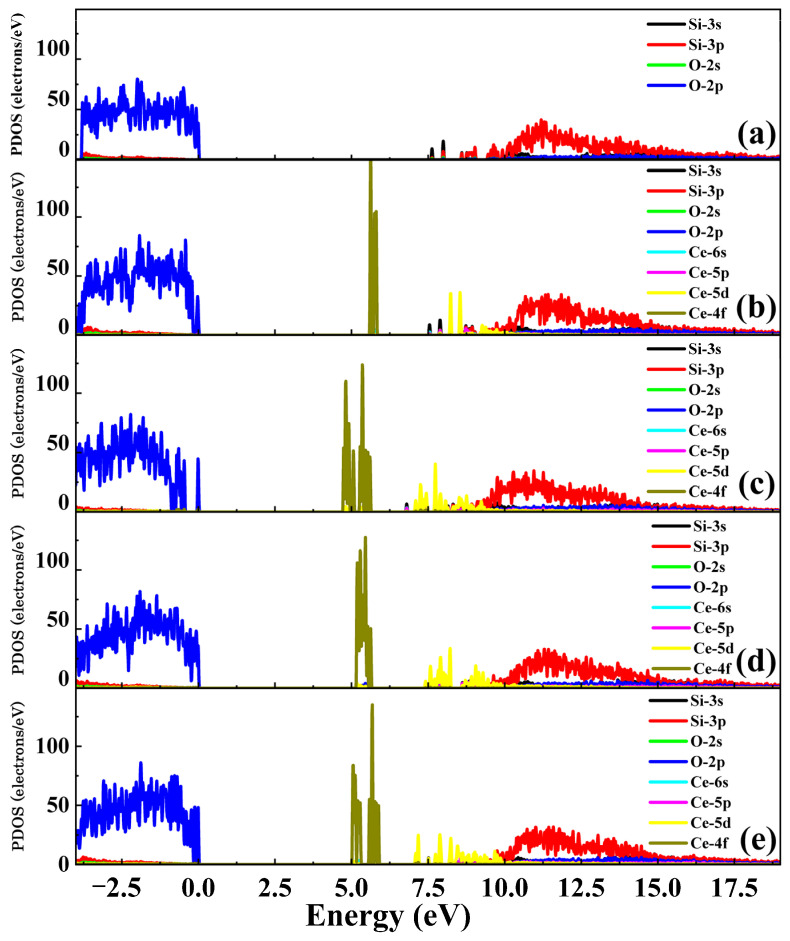
PDOS of intrinsic fused silica and Ce-substitutional doping models: (**a**) intrinsic fused silica; (**b**) Ce vertex substitutional doping; (**c**) adjacent-site substitutional configuration; (**d**) shoulder substitutional configuration; (**e**) opposite substitutional configuration.

The contributions of individual electronic orbitals of Si, O and Ce atoms in the intrinsic fused silica model and cerium-doped models to the band structures of the respective models were derived from partial density of states (PDOS) analysis, as illustrated in Figure A1. For the purpose of facilitating comparison, the Fermi levels of the intrinsic fused silica model and the impurity model presented in the PDOS plots were aligned and set to 0 eV. In the PDOS spectra, the 2p orbital electrons of O atoms predominantly contribute to the conduction band minimum (CBM) within the band structure of intrinsic fused silica (Figure A1a), whereas the 3s orbital electrons of Si atoms primarily account for the valence band maximum (VBM) in the band structure of the intrinsic fused silica model (Figure 4a).

By comparing the PDOS spectra of intrinsic fused silica and Ce-doped fused silica models, we find that Ce doping introduces localized defect states at distinct energy levels within the band gap of intrinsic fused silica, which consequently modulates the energy positions of the VBM and CBM significantly. As illustrated in Figure 4b, single Ce atom substitutional doping at the vertex site slightly downshifts the CBM energy of intrinsic fused silica to 7.49 eV, where the dominant orbital component remains the Si-3s state. In contrast, the three configurations of double Ce atom substitutional doping—adjacent, shoulder, and opposite configurations—downshift the CBM to 7.16 eV, 7.36 eV, and 7.04 eV, respectively. Further analysis reveals that the CBM of the adjacent configuration (Figure 4c) is still dominated by the Si-3s orbital; whereas the CBM downshift in the shoulder (Figure 4d) and opposite (Figure 4e) configurations is primarily driven by the contribution of Ce-5d orbitals. The Ce doping-induced CBM energy downshift and the associated evolution of orbital composition constitute the key mechanism underlying the band gap narrowing of fused silica.

**Figure 5:**
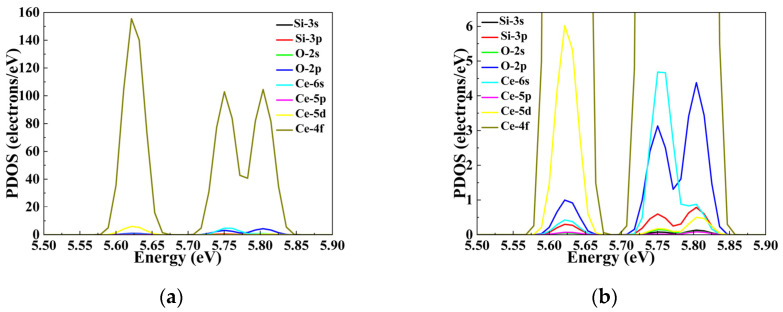
Localized defect states induced by single-Ce vertex substitutional doping in intrinsic fused silica: two defect states appear within the band gap at 5.58–5.68 eV and 5.71–5.86 eV above the VBM, respectively. (**a**) PDOS spectra of the local defect states induced by the substitutional doping of a single cerium atom at the vertex in intrinsic fused silica; (**b**) A local magnification of the defect energy level region corresponding to (**a**).

Across all Ce-containing models, the Ce-4f orbitals serve as the principal contributors to the induced defect states. However, the nature of secondary orbital contributions varies depending on the doping configuration. Consequently, configuration-dependent defect state distribution characteristics are exhibited in the defect band. For the single-Ce vertex-doped model (Figure 5), two defect states appear at 5.58–5.68 eV and 5.71–5.86 eV, contributing to the overall band gap reduction. The lower-energy state (5.58–5.68 eV) arises from hybridization involving all orbitals of Ce and O atoms, as well as the Si-3p orbital, with Ce-5d orbitals exhibiting stronger contributions than other orbitals except Ce-4f. The higher-energy state (5.71–5.86 eV) involves contributions from all atomic orbitals of Ce, Si, and O, and consists of two sub-peaks: the Ce-6s orbital contributes significantly to the first peak but diminishes in the second, whereas Ce-5d contributions increase in the second peak relative to the first.

**Figure 6:**
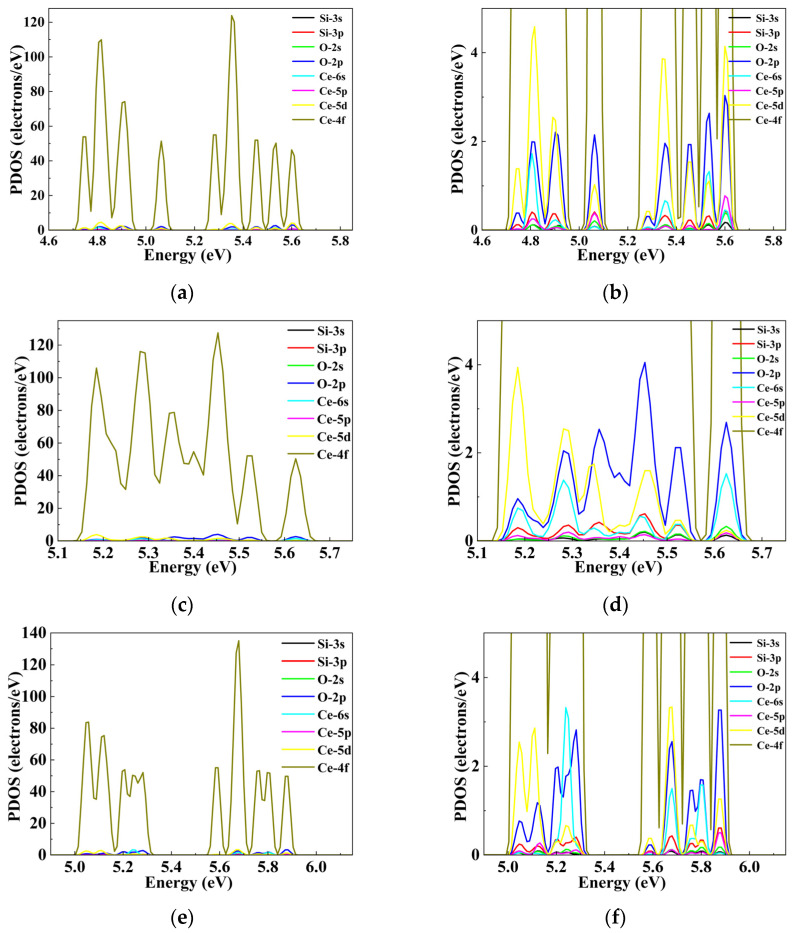
PDOS distribution of defect states induced by double Ce-atom substitutional doping within the band gap of intrinsic fused silica: (**a**) Adjacent-site configuration; (**c**) Shoulder-site configuration; (**e**) Opposite-site configuration. (**b**,**d**,**f**) correspond to the locally magnified PDOS plots of the aforementioned defect states, respectively.

As shown in Figure 6, the three configurations of double Ce substitutional doping (adjacent, shoulder, and opposite) introduce additional Ce atoms, which significantly enhance local orbital interactions and thus induce more abundant and structurally distinct defect states within the band gap of intrinsic fused silica. For the adjacent configuration of double Ce substitutional doping (Figure 6a,b), three energetically isolated defect states are induced within the band gap, centered at 4.7–4.97 eV, 5.02–5.13 eV, and 5.23–5.66 eV, respectively. Within the 4.7–4.97 eV regime, the defect state consists of three well-resolved sub-peaks: The Si-3s, O-2p, and Ce-6s orbitals make no significant contribution to the first sub-peak; The weight of the Ce-6s orbital exhibits a distinct decreasing trend from the second to the third sub-peak; The Ce-4f and Ce-5d orbitals dominate across the entire regime, with the most pronounced contribution at the second sub-peak(~4.80 eV). The 5.23–5.66 eV defect state is composed of five sub-peaks, where the Ce-5d orbital shows the highest orbital weight at the second and fifth sub-peaks. Across all three defect state regimes, the O-2p orbital displays a relatively stable contribution intensity. In the 5.02–5.13 eV regime, its projected weight exceeds that of the Ce-5d orbital but remains significantly lower than that of the Ce-4f orbital.

For the opposite configuration of double Ce substitutional doping (Figure 6c,d), two energetically isolated defect states are induced within the band gap, spanning 5.00–5.34 eV and 5.53–5.93 eV, respectively. The Ce-5d, Ce-6s, and O-2p orbitals act as secondary contributors to the formation of both defect states: Within the 5.00–5.17 eV and 5.62–5.71 eV sub-regimes, the projected weight of the Ce 5d orbital exceeds that of the O-2p orbital; In the 5.18–5.34 eV sub-regime, the projected weights of the Ce-6s and O-2p orbitals are comparable, with their contributions to the defect states being nearly equivalent.

For the shoulder configuration of double Ce substitutional doping (Figure 6e,f), a single defect state spanning 5.13–5.69 eV is induced within the band gap of intrinsic fused silica. Within this defect state: The projected weight of the Ce-5d orbital exhibits a monotonic decay trend with increasing energy (i.e., toward the CBM); The projected weight of the O-2p orbital shows a non-monotonic evolution, peaking at ~5.30 eV before gradually decreasing; The Ce-6s orbital contributes weakly across the entire energy range, and together with the Ce-5d and O-2p orbitals, acts as a secondary contributor to the defect state.

### 3.2. Effects of Ce Doping on the Optical Properties of the Intrinsic Fused Silica Model

**Figure 7:**
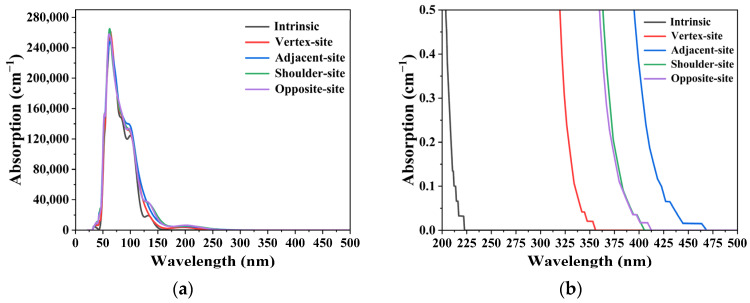
Theoretical Absorption Spectra of Intrinsic and Ce-Doped Fused Silica Models. (**a**) Absorption spectra; (**b**) Magnified view of the absorption edge region, where the absorption edge of the Ce-doped model exhibits a 246 nm red-shift relative to the intrinsic counterpart.

Using the HSE06 hybrid functional with spin-polarization effects considered, optical absorption spectra were calculated for intrinsic fused silica and four representative Ce substitutional doping configurations (vertex, adjacent, shoulder, and opposite-site). A 2 × 2 × 3 k-point grid was employed for Brillouin zone integration, balancing computational efficiency while enabling sufficient resolution of doping-induced local optical responses. The computed absorption spectra are presented in Figure 7: The absorption edge of intrinsic fused silica lies at 222 nm, indicating an extremely low intrinsic absorption coefficient at the 355 nm laser wavelength—rendering it an optical transparency window. In contrast, all Ce-substituted models exhibit a pronounced absorption edge redshift, with cutoff wavelengths of 355 nm, 468 nm, 405 nm, and 412 nm for the vertex, adjacent, shoulder, and opposite-site configurations, respectively. This redshift originates from localized intra-bandgap defect states formed by the hybridization of Ce 4f and O 2p orbitals, which confers the material with substantial light absorption capacity in the 355 nm spectral range. This absorption leads to laser energy deposition at defect sites, exacerbated thermal accumulation, and ultimately triggers local thermal runaway and laser-induced damage.

### 3.3. Fluorescence from Surface-Residual Cerium in Fused Silica Components

**Figure 8:**
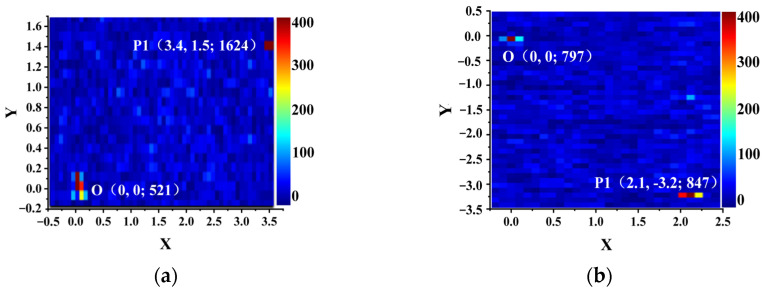
Two-dimensional spatial distribution of localized photothermal weak absorption in the fused silica glass sample. (**a**,**b**) are two-dimensional spatial distribution maps of local PTWA for different samples.

The spatial coordinates and PTWA values of surface defects were obtained through point-by-point scanning using a high-resolution PTWA mapping system. A focused spot size of 20 μm was employed, with a step size of 100 μm, resulting in a measurement grid of 100 × 100 points across the sample surface. The results are presented in Figure 8. In the two-dimensional WPA distribution map, the artificial indentation is denoted as O(0, 0; N), where N represents its absorption coefficient in ppm. Absorption hotspots or regions of elevated signal intensity are labeled as P(x, y; M), with (x, y) indicating the spatial coordinates relative to the origin O (in mm) and M representing the corresponding absorption coefficient (ppm).

**Figure 9:**
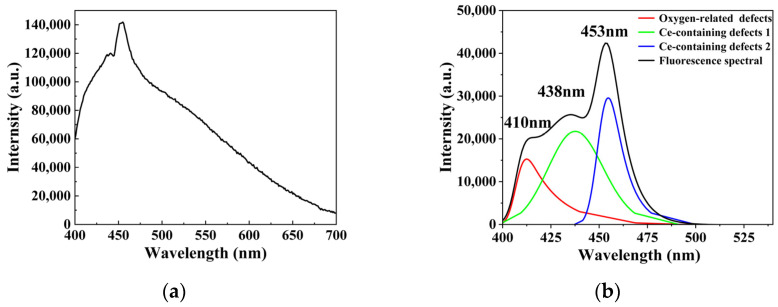
Ultraviolet fluorescence spectrum of the localized contaminated region hosting Ce-associated absorptive defects in the fused silica sample. (**a**) Raw fluorescence spectrum; (**b**) Fluorescence spectrum after multi-peak fitting deconvolution.

**Figure 10:**
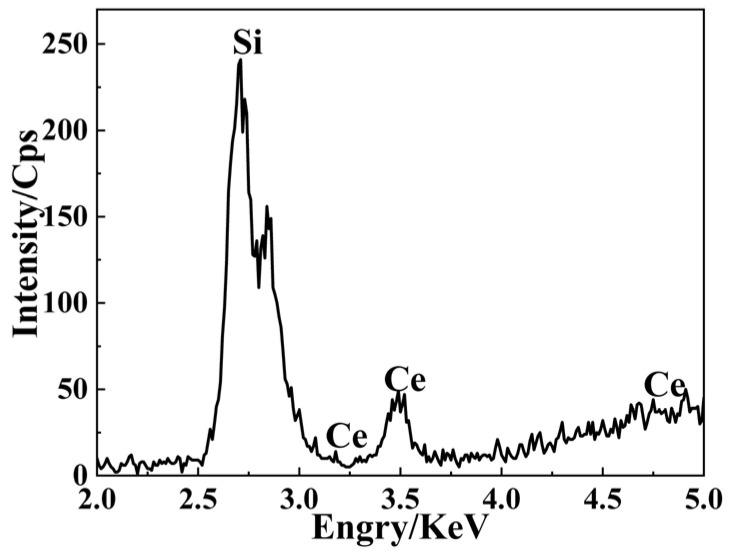
The μ-XRF distribution map of the residual region hosting Ce-associated defects on the surface of fused silica optical components.

**Figure 11:**
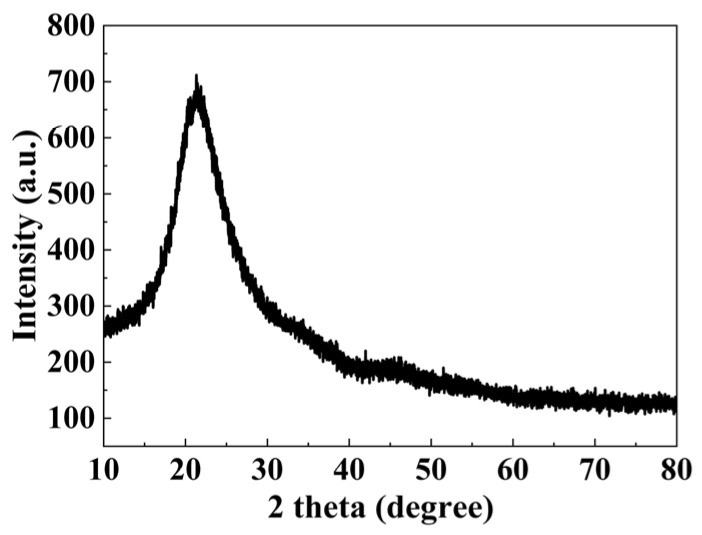
The μ-XRD pattern of the residual region hosting Ce-associated defects on the surface of fused silica optical components.

Ultraviolet fluorescence spectroscopy was conducted on regions exhibiting anomalous PTWA signals, with results presented in Figure 9. Upon excitation at 355 nm, a well-defined fluorescence emission peak centered at approximately 450 nm was observed. To systematically deconvolve the components of the fluorescence spectrum, we performed quantitative analysis of the raw data via multi-peak fitting: The baseline was subtracted using the asymmetric least squares (ALS) method, with parameters set as follows: asymmetry factor λ = 0.001, smoothing factor *p* = 5, and 10 iterations. The baseline-corrected spectrum was fitted with a Gaussian–Lorentzian hybrid function (GLF), yielding three dominant peaks at 410 nm, 438 nm, and 453 nm. Literature comparison and energy-level analysis reveal that: The 410 nm emission peak is associated with typical oxygen defects in fused silica (see [17,18]). The 438 nm and 453 nm peaks are both attributed to localized defect states formed by the adjacent-site doping of double cerium atoms. In Figure 6a, this adjacent configuration introduces three discrete defect energy levels in the band gap: 4.69–4.97 eV, 5.01–5.12 eV, and 5.23–5.66 eV. The energy differences corresponding to the 438 nm (2.83 eV) and 453 nm (2.73 eV) emissions both fall within the lowest subband (4.69–4.97 eV), further confirming their common origin. To elucidate the origin of this luminescence, μ-XRF and μ-XRD analyses were performed on the fluorescent defect regions, with results displayed in Figure 10 and Figure 11. μ-XRD analysis revealed the absence of distinct crystalline diffraction peaks in both the absorptive defect regions and adjacent areas. μ-XRF results demonstrated that only the matrix element silicon (100.00 wt%) was detected in defect-free regions; in contrast, in absorptive defect sites, cerium (0.0212 wt%) was identified alongside silicon (99.9788 wt%). The three-sigma uncertainty associated with the Ce content determined via μ-XRF analysis is 0.0192 wt%. In summary, the experimentally measured fluorescence spectrum can be explicitly decoupled into two contributions: A 410 nm broad-band emission arising from intrinsic oxygen defects in the matrix; 438 nm and 453 nm emissions induced by the adjacent-site doping of double cerium atoms.

**Figure 12:**
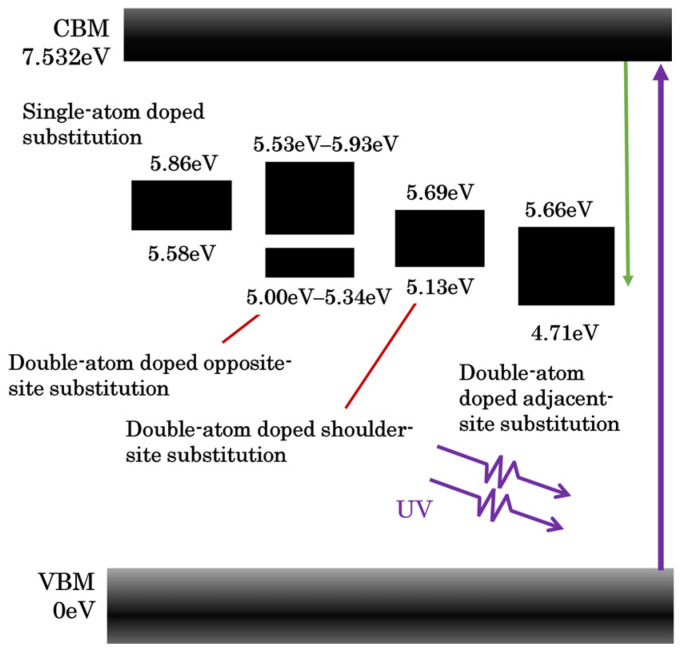
Schematic band structure illustrating the formation of Ce-induced defect states within the band gap of intrinsic fused silica.

Notably, the ~450 nm fluorescence peak experimentally observed under 355 nm laser excitation not only aligns well with the characteristic emission spectrum of Ce-doped fused silica reported in the literature [19], but also precisely matches the radiative transition energy predicted by first-principles calculations. This transition occurs from a 4f-state-based sub-level (4.77–4.85 eV) within the localized defect states (4.71–4.97 eV) induced by adjacent dual-Ce substitution, to a Ce-5d-dominated energy level near the conduction band minimum (CBM, ~7.5 eV, see Figure 4c). The corresponding photon energy difference (2.682–2.762 eV) yields a theoretical emission wavelength range of 448–462 nm, which is in agreement with the experimentally measured fluorescence peak position. Furthermore, the absorption edge red-shifts from 222 nm (intrinsic fused silica) to 468 nm, further verifying that this defect configuration satisfies the band structure prerequisite for light absorption at 355 nm and subsequent ~450 nm fluorescence emission. Based on the above electronic structure calculations, Figure 12 presents a schematic band diagram of the Ce-doped defect site. Under 355 nm laser irradiation, the energy transfer process proceeds as follows: the fused silica matrix adjacent to the Ce-doped site first absorbs photons, exciting valence band electrons to the conduction band. A fraction of the excited electrons transfer energy to the 5d level of Ce^3+^ via non-radiative relaxation, promoting Ce^3+^ to an excited state. Subsequently, Ce^3+^ undergoes a 5d→4f radiative transition, releasing photons and forming ~450 nm-centered fluorescence. The remaining energy is dissipated as lattice vibrations, leading to local temperature elevation—a thermal effect that is highly consistent with the experimentally measured PTWA response. In summary, the experimental observations and theoretical simulations are consistent across three dimensions: spectral position, band structure evolution, and energy transfer pathway. These results fully confirm that the observed ~450 nm fluorescence originates from Ce-doped defects introduced during the polishing process.

LIDT measurements were performed on defect sites exhibiting 450 nm fluorescence emission. The experiment employed a nanosecond pulsed laser operating at a wavelength of 355 nm with a pulse duration of 7.6 ns, and the focused spot area was 330 μm^2^. Multi-pulse cumulative irradiation was conducted following the S-on-1 testing protocol, which entails applying a sequence of constant-energy laser pulses to the identical defect location until irreversible optical damage is detected. The results demonstrated that distinct damage initiation occurred in the defect region when the cumulative fluence exceeded 15.9 J/cm^2^. This value is notably lower than the theoretical LIDT of intrinsic fused silica, thereby confirming that such defects substantially degrade the local laser damage resistance of the material.

## 4. Conclusions

This study explores how substitutional Ce doping modulates the electronic band structure of fused silica. Via MD simulations and first-principles DFT calculations, it is shown that Ce doping narrows the band gap and introduces distinct intergap defect states. Experimentally, fused silica samples processed by conventional ring polishing and acid cleaning were characterized using μ-XRD and μ-UVPL. Results confirm that surface Ce impurities stem from polishing residues, and the 450 nm photoluminescence emission originates from adjacent dual-Ce substitutional defects. Integrated analysis reveals that these Ce-induced defect levels enhance light absorption, triggering localized photothermal heating and heat accumulation. These findings elucidate the mechanism by which polishing-residual Ce degrades the optical performance of fused silica, while providing actionable guidance for optimizing post-processing to improve the reliability of high-power laser systems.

## Data Availability

The original contributions presented in this study are included in the article. Further inquiries can be directed to the corresponding author.

## Appendix A

**Table A1:** Bond Lengths and Bond Angles Between Ce Atoms and Their Neighboring O Atoms in Different Substitutional Doping Models.

Type	Bond Length (Å)					
Single-atom doping of Ce						
Ce–O	2.058	2.091	2.076	2.102		
Double-atom doping of Ce						
Adjacent-site substitutional configuration: Ce–O	2.091	2.192	2.101	2.120		
	2.041	2.182	2.144	2.153		
shoulder configuration: Ce–O	2.069	2.163	2.294	2.077		
	2.077	2.125	2.192	2.094		
opposite substitutional configuration: Ce–O	2.055	2.117	2.195	2.056		
	2.103	2.088	2.08	2.103		
Type	Angle (°)					
Single-atom doping of Ce						
O–Ce–O	100.146	107.319	101.645	126.073	104.644	114.999
Double-atom doping of Ce						
Adjacent-site substitutional configuration: Ce–O	77.363	143.456	107.237	85.552	96.066	76.819
	95.619	156.259	104.224	91.857	97.792	93.238
shoulder configuration: Ce–O	126.018	103.248	93.723	121.984	95.95	110.832
	124.068	103.458	116.638	87.675	118.829	85.53
opposite substitutional configuration: Ce–O	86.724	125.192	123.706	110.701	102.737	100.614
	91.835	95.861	113.73	94.981	149.057	94.551
